# Metabolic Syndrome Mortality in a Population-Based Cohort Study: Jichi Medical School (JMS) Cohort Study

**DOI:** 10.2188/jea.17.203

**Published:** 2007-12-19

**Authors:** Yasunori Niwa, Shizukiyo Ishikawa, Tadao Gotoh, Kazunori Kayaba, Yosikazu Nakamura, Eiji Kajii

**Affiliations:** 1 Division of Community and Family Medicine, Center for Community Medicine, Jichi Medical University.; 2 School of Health and Social Services, Saitama Prefectural University.; 3 Department of Public Health, Jichi Medical University.

**Keywords:** Metabolic Syndrome X, Mortality, Cohort Studies, Japan, Cardiovascular Diseases

## Abstract

**BACKGROUND:**

Metabolic syndrome is known to increase morbidity and mortality of cardiovascular disease. The National Cholesterol Education Program Adult Treatment Expert Panel III in 2001 (revised in 2005) and the Japanese definition of metabolic syndrome were launched in 2005. No study regarding the association between metabolic syndrome by Japanese definition and mortality has been performed. The aim of this study was to clarify the prevalence of metabolic syndrome and its effects to mortality in a population-based cohort study.

**METHODS:**

A total of 2176 subjects who satisfied the necessary criteria for metabolic syndrome were examined between 1992 and 1995 as a part of Jichi Medical School Cohort Study by Japanese definition. Cox's proportional hazard models were used to analyze the association of metabolic syndrome with mortality.

**RESULTS:**

The prevalence of metabolic syndrome was 9.0% in males and 1.7% in females. There were 17 deaths (14 males), including 6 cardiovascular deaths (5 males), during a 12.5-year follow-up period among metabolic syndrome subjects. After adjusting for age, smoking status, and alcohol drinking status, the hazard ratio (95% confidence interval) for all-cause mortality was 1.13 (0.64-1.98) in males and 1.31 (0.41-4.18) in females, and HR for cardiovascular mortality was 1.84 (0.68-4.96) in males, and 1.31 (0.17-9.96) in females.

**CONCLUSION:**

No statistical significant relationship between metabolic syndrome by Japanese definition and all-cause mortality was observed in a population-based cohort study.

Metabolic syndrome is defined as a cluster of multiple risk factors, including central obesity, hypertension, hyperlipidemia, and impaired glucose tolerance, which increases cardiovascular disease morbidity and mortality.^1^^, ^^2^ The third revision of the Adult Treatment Panel, guidelines for cholesterol testing and management in the United States was published by the National Cholesterol Education Program in 2001.^3^ In 2005, the Examination Committee of Criteria for Metabolic Syndrome in Japan^4^^-^^6^ proposed a new set of criteria for the diagnosis of metabolic syndrome. In the same year, the International Diabetic Federation presented a new criterion, which was an essential component, to measure waist circumference with an ethnic-specific value.^7^ Additionally, the American Heart Association / National Heart, Lung, and Blood Institute also modified the National Cholesterol Education Program criteria.^8^

Several studies about the prevalence of metabolic syndrome in the Japanese general population using various criteria have been performed; ^9^^-^^15^ however, there have been few studies of the association with metabolic syndrome by the National Cholesterol Education Program Adult Treatment Panel-III definition and cardiovascular mortality in the Japanese general population,^9^^,^^12^^,^^16^^,^^17^ and Iso H et al and Kadota et al examined using body mass index instead of waist circumferential measurement.^16^^,^^17^ In western countries, Lakka et al reported all-cause mortality and cardiovascular mortality of metabolic syndrome by the National Cholesterol Education Program definition in non-diabetic Finnish middle-aged men,^18^ and several studies^19^^,^^20^ showed that metabolic syndrome in males increased cardiovascular mortality. However, since the Japanese criteria were defined in 2005, to our knowledge, no study about the association between metabolic syndrome by Japanese definition and mortality has been reported.

The purpose of this study was to examine the associations of all-cause mortality, cardiovascular mortality, and the prevalence with or without metabolic syndrome diagnosed by the Japanese criteria for the Japanese general population.

## METHODS

The Jichi Medical School (JMS) Cohort Study is a prospective population-based cohort study to clarify the risk factors of cardiovascular disease in a Japanese rural population. Details on the JMS Cohort Study design and some descriptive data have been published previously.^21^^,^^22^ Baseline data were collected between 1992 and 1995 in 12 rural communities. A total of 12,490 subjects (4,911 males and 7,579 females) participated in the 12 districts, and 2,286 subjects in 3 of these districts had their waist circumference measured (Takasu, Wara, and Sakuma). We excluded 70 subjects who had a previous history of stroke, coronary heart disease, and malignant neoplasm, and 40 subjects from whom we did not obtain a blood sample. Finally, 2,176 subjects (914 males and 1,262 females) were observed in the present study. The participation rate for people invited to the mass screening examination was 56%.^22^

Mass screening examinations for cardiovascular disease have been conducted since 1983 in accordance with the Health and Medical Service Law for the Aged, and we used this system to collect the data. In each community, a local government office sent personal invitations to all the subjects by mail. Trained interviewers using a standardized questionnaire obtained information about their medical history and lifestyle. Smoking status was classified into current smoker, ex-smoker, or never smoked, and alcohol-drinking status was classified into current drinker, ex-drinker, or never drinking alcohol.

Body Mass Index was calculated as weight (kg) divided by the square of body height (m). Waist circumference was measured at the level of the high point of the iliac crest. Systolic and diastolic blood pressures were measured with a fully automated sphygmomanometer, BP203RV-II (Nippon Colin, Komaki, Japan). Serum total cholesterol and triglyceride were measured by an enzymatic method (Wako, Osaka, Japan; interassay coefficient of variation (CV): 1.5% for total cholesterol, and 1.7% for triglyceride). High-density lipoprotein cholesterol was measured by phosphotungstate precipitation (Wako, Osaka, Japan; interassay CV: 1.9%). Plasma glucose was measured by an enzymatic method (Kanto Chemistry, Tokyo, Japan; interassay CV: 1.9%).

Death certificates were obtained from public health centers with permission of the Agency of General Affairs and the Ministry of Health, Labour and Welfare. Each municipal government annually obtained information about subjects who had moved away.

We defined cardiovascular disease as stroke (brain infarction, brain hemorrhage, and subarachnoid hemorrhage), coronary heart disease (angina pectoris and myocardial infarction), and heart failure.

Written informed consent for the study was obtained individually from the responders at the mass screening examination health check-up. We explained that we would gather data using the questionnaire and blood samples, would follow-up their health status, and check the medical records of hospitals if a stroke or myocardial infarction was suspected to have occurred. All responders agreed to join the study. The Institutional Review Board of Jichi Medical School for ethical issues approved this study.

### Metabolic Syndrome

The original diagnostic definition of metabolic syndrome in Japan was presented by the Examination Committee of Criteria for Metabolic Syndrome in April 2005.^4^^,^^5^ Subjects had to satisfy the following criteria: waist circumference 85+ cm for males and 90+ cm for females as an essential component, combined with 2 or more of the following components: (1) triglycerides 150+ mg/dL and/or high-density lipoprotein cholesterol <40 mg/dL; (2) systolic blood pressure 130+ mmHg and/or diastolic blood pressure 85+ mmHg; and (3) fasting plasma glucose 110+ mg/dL. We included subjects treated for diabetes and hypertension by questionnaire at baseline; however, we did not take treatment of hyperlipidemia into account because we did not identify subjects treated for raised total cholesterol, triglyceride, or lower high-density lipoprotein cholesterol by questionnaire.

### Statistical Analysis

All statistical analyses were performed on a personal computer with the Statistical Package for Social Science^®^ (SPSS) for Windows (SPSS Japan Inc., version 11.5, Tokyo, Japan). The results are expressed as the mean ± standard deviation (SD). P-values were calculated using Student's t-test for variables. Smoking status, alcohol-drinking status, and a history of hypertension and diabetes mellitus were tested using the chi-square test.

Cox's proportional hazard models were used to calculate the hazard ratios (HRs) of all-cause mortality and cardiovascular mortality adjusted for age, smoking status, and alcohol-drinking status with or without metabolic syndrome using Japanese criteria. Crude mortality rates were calculated per 1000 person-years.

A p-value less than 0.05 was considered to indicate statistical significance.

## RESULTS

The mean follow-up period (± SD) was 12.5 ± 2.3 years, the total observed person-year was 27,140, and the mean age at baseline ± SD was 56.1 ± 12.2 (56.4 ± 12.4 in males, and 55.9 ± 12.1 in females). There were 220 deaths (10.1%) during the study period: 141 male deaths (15.4%) and 79 female ones (6.2%).

Table 1 shows the data for subjects with or without metabolic syndrome by sex. The prevalence of metabolic syndrome by the Japanese definition was 9.0% in males and 1.7% in females at baseline. There were no significant differences in current smoking and alcohol drinking status between the metabolic syndrome group and the non-metabolic syndrome group, according to the Japanese definition, in both sexes. In females, age was higher in the metabolic syndrome group than in the non-metabolic syndrome group, but in males, there was no significant difference in age between the two groups. Other categories (except high-density lipoprotein cholesterol) were higher in the metabolic syndrome group than in the non-metabolic syndrome group.

**Table 1.** Clinical characteristics of subjects with and without metabolic syndrome.

	with metabolic syndrome	without metabolic syndrome	p-value*
	Males		
n (%)	82 (9.0)	832 (91.0)	
Age (year)	57.8 ± 12.2	56.3 ± 12.4	N.S.
Body Mass Index (kg/m^2^)	26.4 ±2.1	22.4 ± 2.6	< 0.001
Height (cm)	163.7 ± 6.5	162.3 ± 7.2	N.S.
Weight (kg)	70.9 ± 7.9	59.2 ± 9.5	< 0.001
Waist circumference (cm)	90.3 ± 4.7	77.9 ± 7.8	< 0.001
Systolic blood pressure (mmHg)	142.9 ± 17.9	127.4 ± 21.2	< 0.001
Diastolic blood pressure (mmHg)	86.0 ± 11.2	76.8 ± 12.1	< 0.001
Fasting plasma glucose (mg/dL)	108.8 ± 28.0	94.8 ± 15.7	< 0.001
Total cholesterol (mg/dL)	194.7 ± 30.3	185.0 ± 32.9	0.01
HDL cholesterol (mg/dL)	38.4 ± 7.9	49.2 ± 13.6	< 0.001
Triglyceride (mg/dL)	190.2 ± 104.1	111.5 ± 86.0	< 0.001

Current smoking, n (%)	36 (43.9)	405 (48.7)	N.S.
Current alcohol drinking, n (%)	60 (73.2)	632 (76.0)	N.S.
Diabetes Mellitus, n (%)	32 (39.0)	66 (7.9)	< 0.001
Hypertension, n (%)	75 (91.4)	351 (42.1)	< 0.001

	Females		
n (%)	22 (1.7)	1240 (98.3)	
Age (year)	62.0 ± 10.7	55.8 ± 12.1	0.02
Body Mass Index (kg/m^2^)	28.9 ± 4.5	22.8 ± 3.0	<0.001
Height (cm)	148.1 ± 7.7	150.3 ± 6.2	N.S.
Weight (kg)	63.0 ± 8.2	51.5 ± 7.6	< 0.001
Waist circumference (cm)	93.4 ± 3.7	73.7 ± 8.8	< 0.001
Systolic blood pressure (mmHg)	151.0 ± 19.5	130.6 ± 22.4	< 0.001
Diastolic blood pressure (mmHg)	86.4 ± 9.5	76.9 ± 13.1	< 0.001
Fasting plasma glucose (mg/dL)	114.3 ± 35.7	92.3 ± 15.0	< 0.001
Total cholesterol (mg/dL)	212.9 ± 31.9	195.6 ± 33.5	0.02
HDL cholesterol (mg/dL)	41.3 ± 6.5	51.4 ± 11.9	< 0.001
Triglyceride (mg/dL)	172.5 ± 77.5	95.7 ± 51.6	< 0.001

Current smoking, n (%)	1 (4.5)	56 (4.6)	N.S.
Current alcohol drinking, n (%)	7 (31.8)	404 (33.1)	N.S.
Diabetes Mellitus, n (%)	8 (36.3)	78 (6.2)	< 0.001
Hypertension, n (%)	21 (95.4)	598 (48.2)	< 0.001

HDL cholesterol: high-density lipoprotein cholesterol

*: P-values were calculated with Student's t-test for variables and chi-square test for the population.

Data are expressed as the mean ± standard deviation (SD) for variables and percentage for the population.

Table 2 shows the crude mortality rate, cause-specific mortality rate, and HRs calculated by Cox's hazard proportional model with metabolic syndrome, using non-metabolic syndrome as reference. There were 17 deaths (14 males and 3 females) during the follow-up period, and of those, there were 6 cardiovascular deaths (5 males and 1 female). In all-cause mortality, age-adjusted HRs (95% confidence interval [CI]) were 1.05 (0.60-1.82) in males and 1.24 (0.39-3.95) in females. After further adjustment for current smoking and alcohol drinking status, HRs were 1.13 (0.64-1.98) in males and 1.31 (0.41-418) in females. In contrast, age-adjusted HRs of cardiovascular mortality were 1.67 (0.65-4.34) in males and 1.12 (0.15-8.39) in females and all-adjusted HRs were 1.84 (0.68-4.96) in males and 1.31 (0.17-9.96) in females. Our findings suggested that HRs of all-cause and cardiovascular mortality in the metabolic syndrome group were elevated; however, statistical significant differences were not recognized.

**Table 2.** Comparison of adjusted hazard ratios with metabolic syndrome (MetS) by sex.

	Males		Females (90cm) ^‡^		Females (80cm)^§^	
		
	MetS	witout MetS	MetS	witout MetS	MetS	witout MetS
All subjects	914		1262		1262	

MetS subjects, n (%)	82 (9.0)	832 (91.0)	22 (1.7)	1230 (98.3)	95 (7.5)	1157 (92.4)

CVD / Total deaths	5 / 14	28 / 127	1 / 3	25 / 76	1 / 5	23 / 71

All-cause mortality						
Crude mortality (/ 1000 person-years)	14.1	12.4	10.8	4.9	4.0	5.0
HR-Age* (95% CI)	1.05 (0.60 - 1.82)	1.0 (reference)	1.24 (0.39 - 3.95)	1.0 (reference)	0.63 (0.25 - 1.56)	1.0 (reference)
HR-All ^†^ (95% CI)	1.13 (0.64 - 1.98)	1.0 (reference)	1.31 (0.41 - 4.18)	1.0 (reference)	0.52 (0.19 - 1.43)	1.0 (reference)

CVD mortality						
Crude mortality (/ 1000 person-years)	5.0	2.7	3.6	1.6	0.8	1.7
HR-Age* (95% CI)	1.67 (0.65 - 4.34)	1.0 (reference)	1.12 (0.15 - 8.39)	1.0 (reference)	0.37 (0.05 - 2.73)	1.0 (reference)
HR-All ^†^ (95% CI)	1.84 (0.68 - 4.96)	1.0 (reference)	1.31 (0.17 - 9.96)	1.0 (reference)	0.39 (0.05 - 2.94)	1.0 (reference)

CVD: cardiovascular disease

HR: Hazard ratio

CI: Confidence interval

*: Hazard ratios adjusted for age

†: Hazard ratios adjusted for age, smoking status, and alcohol drinking status

‡: Defined as female waist circumference ≥ 90cm

§: Defined as female waist circumference ≥ 80cm

## DISCUSSION

The Japanese diagnostic definition of metabolic syndrome defined waist circumference measurement as an essential component. In 2002, the Japan Society for the Study of Obesity presented new guidelines for obesity, which defined the measurement of waist circumferences as 85+ cm in males and 90+ cm in females.^23^ They presented that the risk of obesity-related disorders (hyperglycemia, dyslipidemia, and hypertension) was increased when the visceral fat area at the umbilical level in Japanese people by CT scan was 100+ cm^2^, and that the waist circumference corresponding to 100 cm^2^ of visceral fat area was 85 cm in males, and 90 cm in females. A characteristic of the Japanese definition is that the cutoff point for female waist circumference is larger than that for males, which is rare in worldwide criteria. Consequently, the Examination of Committee Criteria for Metabolic Syndrome in Japan decided that the component of waist circumference, which is essential for the Japanese diagnostic definition, should be based on the new guidelines for obesity.

Hara et al briefly reported an association with the prevalence of metabolic syndrome defined by the International Diabetes Federation, which required waist circumference measurement for the Japanese general population and recommended appropriate cut-off points of waist circumference of 85+ cm in males and 80+ cm in females.^11^ We reexamined the relationship using the revised waist circumference of 80+ cm, but there were no significant differences between mortality and metabolic syndrome (Table 2).

Recently, several studies reported the prevalence of metabolic syndrome defined by the Japanese definition. Miyatake et al. reported that the prevalence of metabolic syndrome in Okayama Prefecture was 30.7% in males and 3.6% in females.^10^ Urashima et al reported that 14.1% of males and 1.7% of females satisfied the Japanese diagnostic definition of metabolic syndrome.^13^ Arai et al reported that the prevalence of metabolic syndrome by the Japanese definition was 12.1% in males and 1.7% in females, and they suggested that central obesity might be a surrogate marker for metabolic abnormalities. 15 In our study, the prevalence of metabolic syndrome in males using the Japanese definition was about 5-times higher than that in females, and the proportion was similar to the results of other studies.

Several studies have examined associations between mortality and metabolic syndrome in the general population^18^^-^^20^^,^^24^^,^^25^ in western countries. Lakka et al first reported that metabolic syndrome by the National Cholesterol Education Program Adult Treatment Panel III definition in middle-aged non-diabetic Finnish men increased both cardiovascular and all-cause mortality.^18^ Malik et al. also reported that metabolic syndrome in US adults diagnosed by the National Cholesterol Education Program Adult Treatment Panel III increased both overall mortality and cardiovascular mortality.^24^ Katzmaryzyk et al demonstrated that metabolic syndrome in males according to the revised National Cholesterol Education Program definition increased cardiovascular mortality.^25^ There have been few studies about associations between metabolic syndrome and mortality in females in western countries. Qiao et al demonstrated that metabolic syndrome predicted cardiovascular mortality in males, but that the prediction was weak in females.^19^

In Japan, Takeuchi et al. reported that in Japanese men diagnosed with metabolic syndrome by the National Cholesterol Education Program definition during 6-year follow-up, cardiovascular morbidity and mortality were 2.2-times greater than in males without metabolic syndrome.^9^ Kadota et al reported that the prevalence of metabolic syndrome was 18.2% in both sexes by the National Cholesterol Education Program Adult Treatment Panel III definition in NIPPON DATA 90. In this study, total observed person-years were 69,170 and 625 died of all cause and 173 died of cardiovascular disease (27.7%) during a 9.6-year fo low-up period.^17^ Ninomiya et al presented the prevalence of metabolic syndrome was 21% in males and 30% in females with the definition of the revised National Cholesterol Education Program Adult Treatment Panel III in the Hisayama Study, and cardiovascular morbidity of metabolic syndrome was 39.4%.^12^ Kubo et al reported that cardiovascular crude mortality was 2.5 per 1000 person-years in males and 1.4 per 1000 person-years in females in the third cohort of the Hisayama Study.^26^ Crude all-cause mortality was 8.6 per 1000 person-years and crude cardiovascular mortality was 2.4 per 1000 person-years in vital statistics.^27^ The present data are comparable to these.

In our study, there was no significant relationship between metabolic syndrome and mortality using the Japanese definition. Cancer death occupies a third of all-cause mortality in Japan.^27^ Meanwhile, it is said that coronary heart disease is the most frequent cause of death in western countries. The proportion of coronary heart disease mortality among heart disease mortality in Japan is lower than that in western countries; therefore, we considered that the influence on cardiovascular morbidity and mortality was underestimated.^28^

No studies have presented an association between metabolic syndrome and mortality in females; however, we speculated that a waist circumferences 90+ cm as an essential component in the Japanese definition was too strict. In the present study, only 70 females (5.5%) of all subjects satisfied this condition.

The largest limitation of the study is the small sample size. Because of this, the 95% CIs of the HRs are relatively wide. However, we recognize the meaningfulness of the study in spite of the small sample size because seldom longitudinal data considering the metabolic syndrome in Japanese general population exist currently. Continuing the follow-up solve the problem of the small sample size in future.

Our study has several other limitations: (1) waist circumference was measured at the high level of iliac crest; (2) they lived in only 3 rural districts; and (3) drug therapy for dyslipidemia was not identified by questionnaire. However, the methods of measuring waist circumference was not common in the medical health examination when we obtained baseline data for the general population from 1992 through 1995 and various methods of measuring waist circumference are conducted at present. Metabolic syndrome has attracted attention recently in Japan, because waist circumference, which is an essential component, is easy for the general population to measure, but data about metabolic syndrome and mortality in Japanese are not currently sufficient. The Ministry of Health, Labour and Welfare plans to make waist circumference measurement compulsory in medical health check-ups for the general population to monitor and use for the prevention of metabolic syndrome in the future.

In conclusion, there was no significant relationship between metabolic syndrome and all-cause mortality in our present study. We hope that more specific and larger scale prospective studies about cardiovascular events and mortality for the Japanese general population will be performed.
